# Protocol for the practice guideline for traditional Chinese medicine preventive treatment on insomnia disorder

**DOI:** 10.3389/fpsyt.2025.1475904

**Published:** 2025-04-16

**Authors:** Yuting Duan, Yu Wang, Haichun Yang, Yuejuan Cai, Shengwei Wu, Pinge Zhao, Sande Gao, Enyi Liu, Peijing Rong, Lin Yu

**Affiliations:** ^1^ The Affiliated Traditional Chinese Medicine Hospital, Guangzhou Medical University, Guangzhou, China; ^2^ Evidence-based Medicine Center, The Affiliated Traditional Chinese Medicine Hospital, Guangzhou Medical University, Guangzhou, China; ^3^ Sleep Research Institute of Traditional Chinese Medicine, Guangzhou Medical University, Guangzhou, China; ^4^ The Affiliated Guangzhou Hospital of TCM of Guangzhou University of Chinese Medicine, Guangzhou, China; ^5^ Clinical School of Integrated Traditional Chinese and Western Medicine, Guangzhou Medical University, Guangzhou, China; ^6^ Institute of Acupuncture and Moxibustion, China Academy of Chinese Medical Sciences, Beijing, China; ^7^ The Affiliated Brain Hospital of Guangzhou Medical University, Key Laboratory of Neurogenetics and Channelopathies of Guangdong Province and the Ministry of Education of China, Guangzhou Medical University, Guangzhou, China; ^8^ Institute Of Basic Research in Clinical Medicine, China Academy of Chinese Medical Sciences, Beijing, China

**Keywords:** practice guideline, insomnia disorder, TCM preventive treatment, protocol, grade

## Abstract

**Background:**

Most guidelines on insomnia focus more on the treatment of patients after the onset of the illness, with little elaboration on prevention before the onset of the illness. In particular, treatment of insomnia disorder using TCM preventive treatment is rarely mentioned. Therefore, to improve the rehabilitation and quality of life of patients with insomnia disorder and its high-risk groups, the Affiliated TCM Hospital of Guangzhou Medical University and the Institute of Acupuncture and Moxibustion, CACMS have jointly initiated the compilation of the “Practice guideline for TCM preventive treatment of Insomnia disorder”.

**Methods:**

The guideline will adhere to the principles outlined in WHO Handbook for Guidelines Development, 2nd Edition by the World Health Organization and the *Guidelines for the Development of Traditional Chinese Medicine* by the Chinese Society of Traditional Chinese Medicine. The quality of evidence and strength of recommendations will be based on the Grading of Recommendations Assessment, Development, and Evaluation (GRADE) system. The key steps in developing the guideline include: (1) Establishing Guideline Project Group; (2) Conflict of interest management; (3) Research, Selection and Identification of Clinical Questions; (4) Evidence search and evaluation; (5) Quality and grading of evidence; (6) Decision Table for Forming Recommendations; (7) Recommendation Consensus and Determination; (8) Writing the first draft of the guideline; (9) Peer review.

**Discussion:**

Based on the theoretical system of “TCM preventive treatment” of Chinese medicine this guideline is to construct a three-tier preventive framework. The development of this guideline can systematically integrate the characteristic techniques of TCM, such as physical identification, emotional regulation, acupuncture and massage, with the evidence-based evidence of modern sleep medicine, and build a new management model.

**Conclusion:**

This guideline will serve as an evidence-based reference for standardized TCM preventive treatment methods, promoting the standardization and normative application of TCM preventive treatment in insomnia disorder. Ultimately, this guideline aims to improve the rehabilitation level and life quality of individuals with insomnia disorder and those at high risk of the disease.

**Clinical trial registration:**

http://www.guidelines-registry.org/,identifierPREPARE-2023CN517.

## Introduction

Insomnia disorder is a sleep disorder characterized by frequent and persistent difficulty in falling asleep or maintaining sleep, which leads to inadequate sleep quality (1), and accompanied by daytime dysfunction. Nearly one-third of the world’s adults are affected by insomnia disorder (2), with insomnia symptoms or insomnia disorder ranging from 4 to 50%. The prevalence rate of sleep disorders in the elderly population in China is 41.2%, of which the prevalence rates for men and women are 35.7% and 45.0%, respectively, highlighting a gender disparity with a higher rate among females (3). The prevalence of insomnia disorder is higher in women, beginning in adolescence and intensifies during menopause, further amplifying the female predominance (4). Established risk factors includes aging, low socioeconomic status and poor health status or low quality of life (5, 6). Notably, insomnia disorder is often co-morbid with other diseases, including depression, anxiety, hypertension, diabetes, and cardiovascular disease. This comorbidity contributes to significant healthcare costs associated with insomnia (7), underscoring the critical need to address this prevalent Insomnia disorder.

There is a lack of effective premorbid assessment and prevention methods for individuals with insomnia disorder. Insomnia involves complex interactions between psychological cognitive arousal and circadian rhythm and homeostasis mechanism changes (8). Treatment typically involves drug therapy and Cognitive Behavioral Therapy for insomnia(CBTI) to enhance sleep duration and quality, preventing short-term insomnia from becoming chronic. Drug therapy includes benzodiazepine receptor agonists, melatonin receptor agonists and sedative antidepressants, which are more suitable for patients with acute insomnia. While CBTI is the first line of treatment for chronic insomnia (9–11), addressing misconceptions about insomnia and cognitive restructuring to improve sleep. However, limited CBTI-trained professionals, drug side effects, and patient dependency reduce medication adherence and increase recurrence rates.

Traditional Chinese Medicine (TCM) prioritizes preventative measures, aiming to intercept disease before its onset (“preventing illness before it occurs”) (12). This philosophy extends to insomnia management. By identifying individual constitutions through TCM methods, personalized preventive strategies can be implemented. These may include post-meal Tai Chi exercises, Wuqinxi (therapeutic movements), or auricular acupoint therapy during the pre-symptomatic stage (13). In the stage of disease, acupuncture and moxibustion at specific acupoints such as Baihui, Tongtian, Fengchi and other points on the head, and Shenmen, Neiguan and Sanyinjiao can be employed to nourish heart and tranquilize mind. Additionally, TCM foot bath offers a non-invasive option for inpatients. Post-recovery, TCM interventions like sleep sachets or herbal remedies aim to regulate constitution and solidify sleep patterns, minimizing recurrence (14) and enhancing overall well-being (15).

In 2016, Clinical practice guidelines (16) treat insomnia based on dialectical treatment, reflect the individualized medication. In 2017, Clinical Practice Guideline (9) gave specific recommendations for the treatment of chronic insomnia in adults. Clinical Practice Guideline (17) published in 2021 and Clinical practice guideline for integrated traditional Chinese and Western medicine rehabilitation of insomnia (18) published in 2025 provided guidance for behavioral and psychological treatments of adult chronic insomnia disorder. The above guidelines all explain the definition, diagnostic criteria, intervention and management of insomnia. Most of them are for the treatment during or after the onset of the disease. There are few recommendations for the intervention of TCM preventive treatment before the onset of the disease, and the content of preventive treatment of disease has not been regularly updated.

Therefore, combined with the international diagnostic criteria and norms on insomnia disorder, absorbing the current research results and successful experience of traditional Chinese medicine in the prevention and treatment of insomnia, drawing on the research methods of clinical epidemiology, forming a clinical practice guideline for the prevention and treatment of insomnia disorder with evidence-based medical evidence is of great significance for improving the prevention and treatment of insomnia in the world and reducing the health and economic burden.

## Methods

This clinical diagnosis and treatment guideline will refer to the relevant requirements of the WHO Guidelines Development Manual compiled by WHO and the TCM Guideline Development Program developed by the China Association of Chinese Medicine, and apply tools such as AGREE II (19) (Appraisal of Guidelines for Research and Evaluation II) and AMSTAR 2 (20) (A Measurement Tool to Assess Systematic Reviews 2)., in order to evaluate the quality of evidence. Body of evidence grading criteria GRADE (21)(Grading of Recommendations Assessment, Development and Evaluation) will also be applied and the guidelines will be compiled and developed in accordance with the RIGHT-TCM (22, 23) (Reporting Items for Practice Guidelines in Healthcare-Traditional Chinese Medicine). The specific workflow is detailed in **Supplementary Material 1**.

### Guidance sponsorship and support units

This guideline will be co-sponsored by the Affiliated TCM Hospital of Guangzhou Medical University and the Institute of Acupuncture and Moxibustion, China Academy of Chinese Medical Sciences, with methodological support from the Institute of Health Data Science at Lanzhou University.

### Trial registration

The guideline has been registered in English and Chinese at the Practice guideline REgistration for transPAREncy platform (PREPARE). The registration number is PREPARE-2023CN517.

### Target population for implementation of the guideline

Patients with insomnia disorder (primary insomnia, psychophysiological Insomnia and adjustment insomnia, excluding children’s sleep disorder and women’s sleep disorder at special times, as well as insomnia caused by definite internal medical, psychiatric, and neurological diseases) and those at high risk.

### Guideline users

Clinicians of relevant departments, such as TCM preventive treatment Department, Psychiatry Department, Clinical Psychology Department, Traditional Chinese Internal Medicine Department, Integrated Chinese and Western Medicine Department, and Community Health Service Center.

### Guideline expert group

The Guidelines Development Group will consist of the Guideline Steering Committee, the Guideline Consensus Expert Group, the Guideline Secretarial Group, the Guideline Evidence Evaluation Group, and the Guideline External Review Expert Group. The specific memberships and responsibilities are listed as below.

### Guideline steering committee

The Guideline Steering Committee will be composed of 9 experts(**Supplementary Material 2**). Main responsibilities: (1) Determine the topic and scope of the guideline; (2) Organize other guideline working groups and manage their conflicts of interest; (3) Review and approve protocol; (4) Oversee the guideline development process; (5) Approve the publication of the recommendation and guideline. The Guideline Steering Committee has three chairs, two of them are clinical specialty chairs and the other one is a methodology chair, and none of them has a conflict of interest related to the guideline.

### Guideline consensus expert group

Based on the consideration of geographical representation and gender, the Guideline Consensus Expert Group will consist of 20 experts in TCM preventive treatment Department, Encephalopathy Department, Psychiatry Department, and Department of Methodology for Evidence-Based Medicine. Main responsibilities: (1) Formulating and reviewing clinical questions and selecting outcomes; (2) Voting and consensus on recommendations; (3) Finalizing the full text of the guideline; In addition, the Guideline Consensus Expert Group will invite 1~2 patient representatives from the daily treatment patients to review the guideline manuscript. The expert group will provide a professional interpretation of the guidelines for the patients, and invite the patient representatives to provide comments and feedback on the summarized evidence and the formed recommendation using the perspective and identity of the ordinary patient.

### Guideline secretarial group

The Guideline Secretarial Group will be composed of the staff of the Affiliated TCM Hospital of Guangzhou Medical University, which is the lead organization of the guideline. Main responsibilities: (1) Coordinate the work of other working groups; (2) Draft the guideline protocol; (3) Conduct investigate and research on clinical questions; (4) Organize consensus meetings on recommendations; (5) Document the whole process of guideline development; (6) Write the first draft of the guideline; (7) Submit the guideline for publication.

### Guideline evidence evaluation group

The Guideline Evidence Evaluation Group will be composed of professionals in evidence-based medicine from all units involved in guideline development who have been engaged in guideline evidence evaluation for at least 1 year. Main responsibilities: (1) Search, evaluate, synthesize, and grade evidence;(2) Create an summary of findings table (24) and a recommendation decision table.

### Guideline external review group

The Guideline External Review Group shall be composed of 3-5 experts who have no conflict of interest with the guideline and are research in guideline-related fields. The main responsibilities: (1) Review of the finalized guideline in its entirety; (2) Propose specific comments and suggestions.

### Conflict of interest management

All members of the Guideline Steering Committee, the Consensus Expert Group, the Guideline Secretarial Group, the Guideline Evidence Evaluation Group will be required to declare all potential conflicts of interest prior to formal participation in the development process or attendance at a meeting and completing a collecting disclosures of interest (COI) form, which includes both financial and non-financial aspects, to be retained by the Guidelines Working Group, and to The COI should be retained by the Guideline Working Group and agreed to be published in the Guideline. The declaration will include the absence of any commercial, professional or other interest in the subject matter of the guideline and any interest that may be affected by the results of the recommendations. If there is a relevant conflict of interest, it will be dealt with in accordance with the “COI management” (see **Supplementary Material 3** for details); if there is no interest to be declared, the declaration of interest form should also be marked “No Interest”.

### Clinical questions

In the early stage, the Guideline Evidence Evaluation Group will use “insomnia disorder” and “TCM preventive treatment” as the search terms to review the relevant literature in the Chinese and English databases, and at the same time, read textbooks and books as well as consult the websites of relevant societies to collect information; In addition, design clinician and patient questionnaires (**Supplementary Material 4**) and complete distribution, retrieval and collation. Immediately, experts in various fields such as encephalopathy, psychiatry, statistics, evidence-based medicine, and patient representatives will be invited to convene two or three rounds of clinical questions formulation and outcomes selection meetings to construct the item pool of relevant basic questions, clinical questions, and outcomes.

Formulating clinical questions: The first and second rounds will be scored on a 4-point scale, including the words “completely disagree”, “relatively disagree”, “relatively agree”, and “strongly agree”. The I-CVI (content validity index) will be calculated for each item, which is the number of experts who “relatively agree” and “strongly agree” for each item divided by the total number of experts participating in the evaluation, is the corresponding item level I-CVI, and requires I-CVI ≤ 0.78 as the exclusion criterion for selection (25). The final list of clinical questions will be validated in the third round using the nominal group method, with≥70% “yes” votes for inclusion.

Selecting outcomes: The importance ranking of the outcomes will be based on a 9-point scale, with 7 to 9 indicating critical to decision-making and recommendation (key outcome), 4 to 6 indicating important to decision-making and recommendation (important outcome), and 1 to 3 indicating of general importance to decision-making and recommendation (general outcome), with a total of two rounds of investigation and research work, and the confirmation of the outcomes will be completed in the third round.

### Evidence search and evaluation

Evidence search: The Patient, Intervention, Comparison, and Outcome (PICO) framework will be constructed for each guideline key question, identifying the corresponding Chinese and English subject terms and free terms, respectively, and developing a search formula based on the search strategy of each data platform. Systematic search of databases such as PubMed, Embase, The Cochrane Library, Epistemonikos, Wanfang Data, China Knowledge Network (CNKI), and China Biomedical Literature Database (CBM) databases, and supplement with searching references from clinical trial registration platforms and retrospectively incorporated literature. The search will be conducted from the time of database construction to February 2025. Literature types include systematic reviews and original studies (randomized controlled trials, clinical trials, diagnostic trials, cohort studies, case-control studies, cross-sectional studies, and case series). An example of the search for “efficacy of acupuncture in the treatment of insomnia disorder” is shown in **Supplementary Materials 5**, **6**.

Evidence screening and data extraction: The Guideline Evidence Evaluation Group will conduct the search independently in groups of 2, and refer to the process shown in **Supplementary Material 7** for the retrieved literature. Initial screening by title, abstract, combine with the literature inclusion and exclusion criteria for re-screening, and then the remaining literature for the full-text reading, different opinions will be resolved by discussion or negotiate to determine by consultation with a third party, to arrive at the final number of literatures included.

Literature quality assessment: The Guideline Evidence Evaluation Group will assess the quality of the evidence separately, with assessment tools including AGREE II, AMSTAR 2, MINORS, CASP checklist, etc. The Guideline Evidence Evaluation Group will develop criteria for grading the quality of the evidence, and the assessment process will be done independently by the Guideline Evidence Evaluation Group in groups of 2, with any disagreements resolved through joint discussion or consultation with a third party, and low-quality evidence will be excluded. Randomized controlled trials, clinical trials, diagnostic trials, cohort studies, case-control studies, cross-sectional studies, and case series will be conducted using the Cochrane Risk of Bias Assessment Tool 2.0, ROBINS-I Tool (Risk of Bias In nonrandomized Studies-of Interventions), QUADAS-2 Tool (Quality Assessment of Diagnostic Accuracy Studies Quality of evidence), NOS scale (Newcastle-Ottawa Scale), AHRQ scale (Agency for Healthcare Research and Quality), and the Canadian Institute of Health Economics IHE Quality Assessment Tool.

Systematic review development and updating: If a key question has a high-quality systematic review within three years, it is directly included; if a high-quality systematic review has been published for more than three years, the high-quality systematic review is updated (26); and if the quality of the systematic review is too low or there is no relevant systematic review, the Guideline Evidence Evaluation Group develops new systematic review to complement the evidence in the guideline, as detailed in the process described in **Supplementary Material 8**.

### Grading of evidence quality

The GRADE (Grading of Recommendations Assessment, Development and Evaluation) (**Table 1**) will be used to grade the quality of evidence for each clinical question, and grade the quality of evidence based on the results of the evaluation (27) and summarize the evidence summary table. Randomized controlled trials are initially considered high-quality evidence, and observational studies are initially considered low-quality evidence, adjusted for downgrading and upgrading factors. Five downgrading factors included study limitations, imprecision, inconsistency, indirectness, and the presence of possible publication bias; three upgrading factors included large effect sizes, the presence of a dose-response, and confounding factors.

**Table 1:** Certainty of evidence and strength of recommendation grading citeria used in this guideline.

Classification	Explicit description
Certainty of evidence	
High (A)	We are very confident that the true effect lies close to that of the estimate of the effec.
Moderate (B)	We are moderately confident in the effect estimate. The true effect is likely to be close to the estimate of the effect, but there is a possibility that it is substantially different.
Low (c)	Our confidence in the effect estimate is limited. The true effect may be substantially different from the estimate of the effect.
Very low (D)	We have very little confidence in the effect estimate. The true effect is likely to be substantially different from the estimate of effect.
Recommended Strength	
Strongly Recommended/Strongly Not Recommended	Clearly show that the intervention does more good than harm or more harm than good
Weakly Recommended/Weakly Not Recommended	Uncertainty about the benefits and disadvantages or evidence of equal quality of benefits and disadvantages

### Formation of recommendations

Possible recommendations will be formed based on the quality of evidence, balance of benefits and drawbacks, combined with patient preferences and values, and cost-effectiveness analysis. The recommendations will be classified into strong and weak recommendations (**Table 1**). Members of the Guideline Consensus Expert Group will reach consensus (75% or more are considered to have reached consensus) on the recommendations through two to three rounds of the Delphi method, agreement and the reasons for disagreement, as well as the consensus process, will be documented in detail and ultimately presented in the appendix. The revised recommendations will be submitted to the Guideline External Review Group for evaluation of the clarity of the guideline and the feasibility of clinical application, etc. Feedback will be collected, organized and reviewed by the Guideline Steering Committee, and appropriate ones will form the final version of the recommendations.

### Reporting and publishing the guideline

Once the final recommendations are formulated, the Guideline Secretarial Group will write the full text of the guideline in accordance with the RIGHT-TCM items (28), and submit it to the Guideline Consensus Expert Group for revision. The Guideline Steering Committee will finalize, approve, and publish it.

### Dissemination, implementation and evaluation of the guideline

After the publication of the guideline, it will be introduced and disseminated through the self-media of the Affiliated Hospital of Traditional Chinese Medicine of Guangzhou Medical University, the Affiliated Brain Hospital of Guangzhou Medical University and related societies and academic conferences. The implementation of the guideline will be carried out through multi-center, large-sample, randomized controlled trials to verify and evaluate the clinical guidance value of the guideline.

### Updating the guideline

This guideline is proposed to be updated once every 3 years (the timing may be adjusted to take into account the latest evidence), and the content of the update depends on whether new, high-quality medical evidence of relevance has appeared since the guideline was published, and whether there is an impact of the change in the evidence on the strength of the guideline’s recommendations; in addition, changes in patients’ wishes or demands will be collected during clinical practice. In the event of objections raised by the current or proposing revision team, a revised methodology plan will be presented to the organizing group or institutional platform for review and modification of the guideline. If no proposal is put forth near the review date, the lead organization will undertake the guideline update. This process will adhere to the internationally published Check List for the Reporting of Updated Guidelines (29), involving steps such as identifying new scientific evidence, evaluating the need for updates, revising guideline recommendations, and publishing the updated guideline.

## Discussion

Insomnia has shown a trend of high incidence and wide population. It has a wide impact on the physical and mental health, memory, innovative function and social activity function of the public (30), so it is necessary to prevent insomnia. Insomnia is related to constitution. Qi deficiency, Yang deficiency and blood stasis are the main constitutions of female insomnia patients, while damp-heat, Yang deficiency and phlegm-dampness are the main constitutions of male insomnia patients. Preventive treatment is a cornerstone of TCM, encompassing both primary prevention (“preventing illness before it occurs”) and secondary prevention (“preventing recurrence after it has healed”). Personalized strategy adjustment serves as the foundation for this approach. Through the implementation of acupuncture, moxibustion, topical formulations, herbal therapies, dietary modifications, and tailored exercise regimens, TCM practitioners address individual.

Susceptibility (31), This comprehensive strategy aims to enhance the body’s resilience, thereby mitigating the onset and relapse of insomnia.

Preventive and therapeutic measures for insomnia. “Chai Hu Long Gu Mu Li” Soup is very effective in preventing patients with qi depressive insomnia (32). The use of music therapy of TCM preventive treatment to regulate emotions and prevent the influence of emotions on insomnia. On the basis of yin and yang and five elements, the Inner Canon of Huangdi combines five tones, five zang organs and five wills to create a music therapy that can cure diseases, which is called five elements music. Five-tone therapy can regulate emotions and the functions of zang-fu organs and meridians, eliminate diseases and strengthen the body, so as to harmonize people’s yin and yang (33). Studies have shown that when the frequency, intensity and rhythm of music are consistent with the physiological rhythm of the human body, a harmonious resonance can be formed, thereby achieving the effect of sedation and help sleep (34). Some studies have used music relaxation to treat elderly patients with insomnia. After 10 days, the effective rate of the experimental group is 80%, which is significantly higher than that of the control group (8%) (35). And music therapy is non-toxic and harmless, economic and safe, through the combination of constitution identification, to achieve the unity of mind, can effectively prevent the occurrence of insomnia.

Pathological changes that occur in various parts of the body are reflected in the Auricular points.

Modern medicine shows that the auricle is rich in lymph, blood vessels, and nerves and intertwine with each other to form a network. Through the auricular acupuncture points, the acupoints are stimulated, causing the normal order of the brain reticular structure. stimulation, causing the normal orderly activation and inhibition of the brain reticulum structure, thus making the pathological sleep state to physiological sleep state. Guixiang Wen research team found that Applying Wang Bu Liu Xing to the subcortex of both ears, heart, Shen Men, sympathetic and other acupoints to prevent preoperative insomnia has significant therapeutic effect (36). For patients who have been diagnosed with insomnia, they can be diagnosed and treated according to the syndrome differentiation of “Insomnia TCM Clinical Practice Guidelines (WHO/WPO)”.

Compared with other interventions, there are several significant advantages of TCM preventive treatment. 1. Holistic view, treating both symptoms and root causes. TCM preventive treatment believes that insomnia is a manifestation of dysfunction of the internal organs (such as the heart, liver, kidneys and spleen) or disharmony of qi and blood, rather than a simple “sleep problem”. 2. Individualized diagnosis and treatment. Chinese medicine practitioners provide personalized solutions based on the patient’s constitution, symptoms and causes (e.g. heart-kidney incompatibility, liver depression and fire, heart-spleen deficiency, etc.). 3. Low side effects, suitable for long-term conditioning. Chinese medicine (natural herbs are the mainstay), with lower dependence compared to Western medicine, suitable for long-term conditioning of chronic insomnia patients.

The guideline is a clinical practice guideline with the theme of “insomnia”. The preliminary drafting plan can not only make the guideline drafting group familiar with the guideline developing process, but also standardize and transparent the work process, so as to improve the quality of this evidence-based application guideline and better serve patients (37).

## Strengths and limitations of this study

1. There is no comprehensive guideline for the diagnosis and treatment of insomnia disorder using TCM preventive treatment, and such a guideline in needed.
2. Combined with the international diagnostic criteria and norms on insomnia disorder, absorbing the current research results and successful experience of traditional Chinese medicine in the prevention and treatment of insomnia, drawing on the research methods of clinical epidemiology, forming a clinical practice guideline for the prevention and treatment of insomnia disorder with evidence-based medical evidence.
3. It puts more emphasis on preventing disease, non-pharmacological therapies are evidence-based and optimized, and multimodal interventions are synergistic.
4. The inclusion of relevant literature published in Chinese and English.

## Conclusion

This guideline intends to clarify the indications of different intervention measures and explore the advantages and disadvantages of different modes of combined application through comprehensive evidence retrieval, standardized evidence synthesis and evaluation, and scientific recommendation formation process, so as to standardize and guide the clinical application of preventive treatment of disease technology in TCM preventive treatment, reduce the occurrence of insomnia and prevent it from aggravation.
