# Supplementary figures and images for: Protocol for the practice guideline for traditional Chinese medicine preventive treatment on insomnia disorder
Source: Front Psychiatry. 2025 Apr 16;16:1475904. doi: 10.3389/fpsyt.2025.1475904 (PMC12041864; doi:10.3389/fpsyt.2025.1475904)

# Supplementary material 1. Technical road map for the development of the guidelines

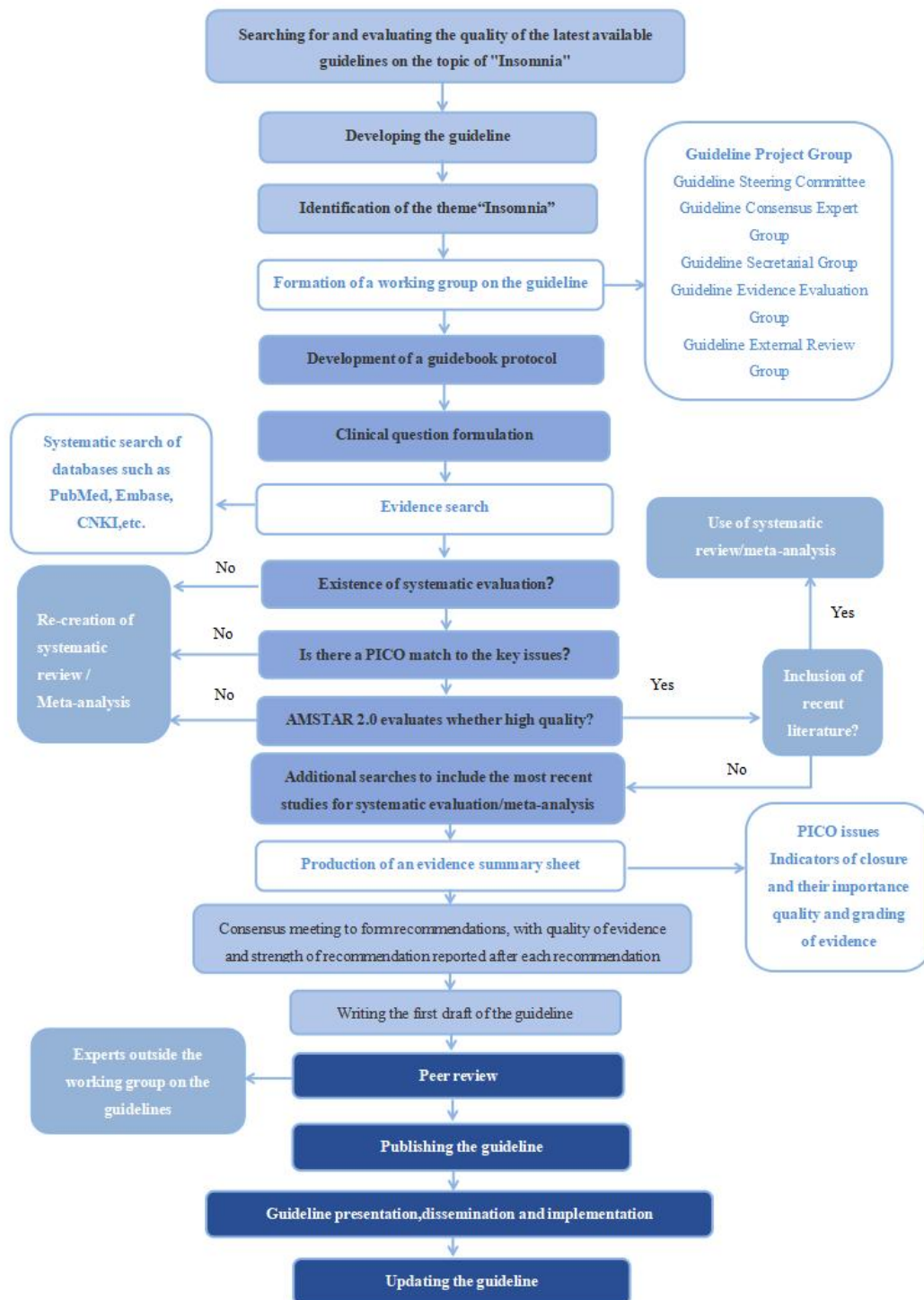

Supplement: Supplementary file 1 [file DataSheet1.pdf]

Supplementary material 7. Flow chart of literature screening

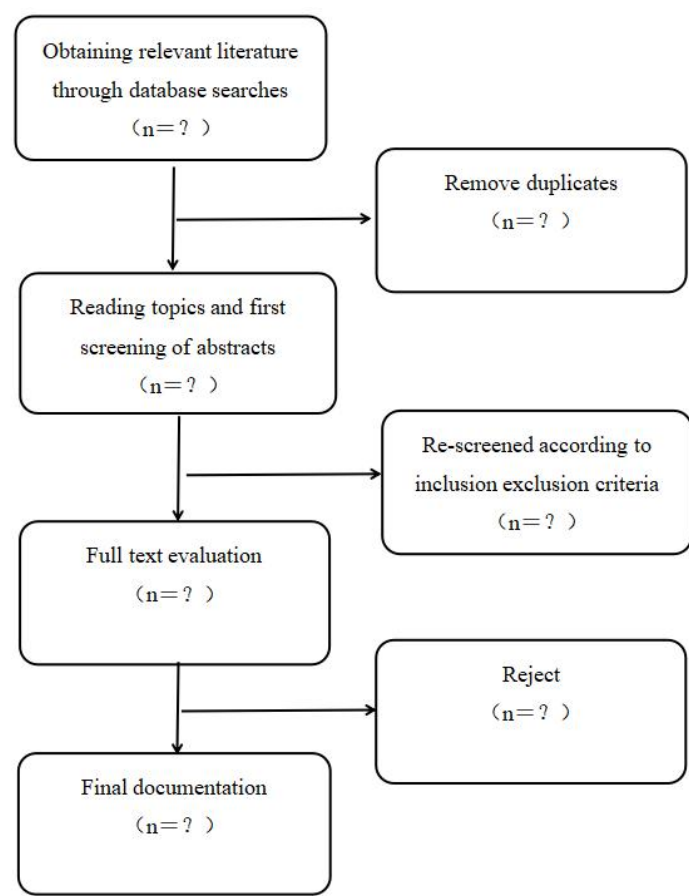

Supplement: Supplementary file 7 [file DataSheet7.pdf]

Supplementary material 8. Flow chart of specific search and screening for system evaluation

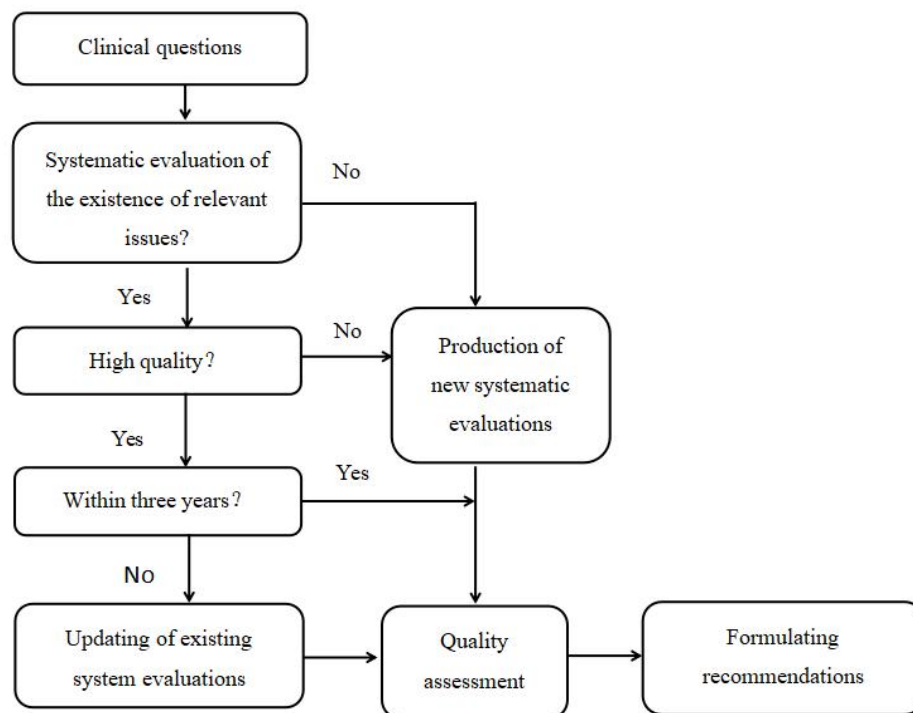

Supplement: Supplementary file 8 [file DataSheet8.pdf]
