# Supplementary material for: Protocol for the practice guideline for traditional Chinese medicine preventive treatment on insomnia disorder
Source: Front Psychiatry. 2025 Apr 16;16:1475904. doi: 10.3389/fpsyt.2025.1475904 (PMC12041864; doi:10.3389/fpsyt.2025.1475904)
Supplement: Supplementary file 2 [file DataSheet2.pdf]

Supplementary material 2. Committee list

Guideline Steering Committee

Yuping Ning, The Affiliated Brain Hospital of Guangzhou Medical University
Hongxiao Jia, Beijing Anding Hospital, Capital Medical University
Lanying Liu, Shanghai Mental Health Center
Lin Yu, The Affiliated Traditional Chinese Medicine Hospital, Guangzhou Medical University
Jianxiong Guo, The Affiliated Brain Hospital of Guangzhou Medical University
Jianguo Shi, Xi 'an Mental Health Center
Na Zhao, The First Affiliated Hospital of Harbin Medical University
Xiaoshan Zhao, School of Traditional Chinese Medicine, Southern Medical University
Lingling Yang, Henan Provincial Hospital of Traditional Chinese Medicine

Guideline Consensus Expert Group

Yan Li, Linhui Zhou, Xinyu Ge, Wenjing Liu, Zhuowei Huang,
Yongjun Chen, Hualing Cai, Huipeng Lu, Zhangjin Zhang, Shengju Li,
Xiang Zhang, Ying Liao, Zhenjuan Qin, Shengzhong Weng, Jie Dong
Shujuan Hou, Guolu Ma, Fei Li, Jingru Cheng, Songxing Zhang

Guideline Secretarial Group

Yuting Duan, Haichun Yang

Guideline External Review Group

Pinge Zhao, Yuejuan Cai, Jin Zhang, Yuening Deng
