# Supplementary material for: Protocol for the practice guideline for traditional Chinese medicine preventive treatment on insomnia disorder
Source: Front Psychiatry. 2025 Apr 16;16:1475904. doi: 10.3389/fpsyt.2025.1475904 (PMC12041864; doi:10.3389/fpsyt.2025.1475904)
Supplement: Supplementary file 3 [file DataSheet3.pdf]

Supplementary material 3. Conflict of interest approach

Level of conflict of interest	Methods of management
No conflict of interest	Will not be a source of bias and does not need to be addressed further, only declarations of interest and evaluation results need to be made public at meetings before formal work begins and reported in the final version of the guidelines
Conflicts of interest exist only in specific areas or aspects of the development of the guidelines	Exclude participants from these specific areas or segments and allow them to participate in areas that are not relevant to the conflict of interest
Conflicts of interest at all stages of guideline development	Termination of participants
