# Supplementary material for: Protocol for the practice guideline for traditional Chinese medicine preventive treatment on insomnia disorder
Source: Front Psychiatry. 2025 Apr 16;16:1475904. doi: 10.3389/fpsyt.2025.1475904 (PMC12041864; doi:10.3389/fpsyt.2025.1475904)
Supplement: Supplementary file 4 [file DataSheet4.pdf]

Supplementary material 4. Issue entry

1. The clinical problems to be paid attention to in the types, methods and selection of intervention techniques in the Traditional Chinese Medicine Preventive Treatment.

(1) What is the role of herbal and proprietary medicines alone in insomnia disorders?

(2) What is the effectiveness and safety of Chinese herbal medicines and proprietary Chinese medicines used alone or in combination for the preventive treatment of patients with insomnia or people at high risk of insomnia, compared with other treatments, in terms of improvement of clinical symptoms, enhancement of the quality of life, and maintenance of long-term therapeutic efficacy?

(3) Can Chinese herbs or proprietary Chinese medicines for insomnia serve to withdraw western medications?

2. Clinical issues to be concerned with non-pharmacological therapies for the Traditional Chinese Medicine Preventive Treatment.

(1) What is the role of acupuncture alone in the preventive treatment of insomnia disorders?

(2) How effective and safe is acupuncture alone or in combination with other treatment modalities in the preventive treatment of insomnia?

(3) How economical is the preventive treatment of insomnia with the Traditional Chinese Medicine Preventive Treatment methods such as acupuncture?

(4) How effective are hypnotherapy and balms in preventing and treating insomnia?

(5) How effective are tuina, gua sha, auricular acupuncture, and therapeutic food treatments in preventing and treating insomnia?

3. Clinical issues to focus on for effective outcome indicators in the field of insomnia Chinese medicine for the Traditional Chinese Medicine Preventive Treatment.

(1) Which outcome indicators are of significant value and representative for the field of insomnia TCM Preventive treatment? For example, the Pittsburgh Sleep Quality Index Scale (PSQI).

(2) Which outcome indicators are more actionable for the field of insomnia TCM Preventive treatment?

4.Other clinical issues that you think need attention.
