# Supplementary material for: Protocol for the practice guideline for traditional Chinese medicine preventive treatment on insomnia disorder
Source: Front Psychiatry. 2025 Apr 16;16:1475904. doi: 10.3389/fpsyt.2025.1475904 (PMC12041864; doi:10.3389/fpsyt.2025.1475904)
Supplement: Supplementary file 5 [file DataSheet5.pdf]

Supplementary material 5. Chinese Search strategies

#1 "sleepless"[theme] OR "dead set in one's eyes"[theme]OR "be unable to sleep"[theme]
#2 "Disorders of sleep initiation and maintenance"[theme] OR "sleep disorder"[theme]OR "insomnia"[theme] OR "chronic insomnia"[theme]OR "difficulty in sleeping"[theme] OR "Circadian rhythm sleep-wake disorders"[theme]OR "hypersomnia"[theme] OR "Excessive daytime sleepiness"[theme]
#3 #1 OR #2
#4 "Acupuncture"[Title/ Keywords/Abstract] OR " Acupuncture Therapy"[MeSH Terms] OR "Acupuncture Treatment"[Title/Abstract] OR "Electroacupuncture"[Title/Abstract]OR "Umbilical acupuncture"[Title/Abstract] OR "Ear Acupuncture"[Title/Abstract] OR "Auricular Acupuncture"[Title/Abstract] OR "scalp acupuncture"[Title/Abstract] OR "abdominal acupuncture"[Title/Abstract] OR "Eye Acupuncture"[Title/Abstract] OR "Buccal acupuncture"[Title/Abstract] OR "arm acupuncture"[Title/Abstract] OR "wrist-ankle acupuncture"[Title/Abstract] OR "body acupuncture"[Title/Abstract]
#5 "Blade acupuncture"[Title/Abstract] OR "filiform needle"[Title/Abstract]OR"thumbtack needle"[Title/Abstract] OR " fire needle"[Title/Abstract] OR "bee acupuncture "[Title/Abstract] OR " plum blossom needle"[Title/Abstract] OR " silver needle"[Title/Abstract]
#6 #4 OR #5
#7 #3 AND #6
