# Supplementary material for: Protocol for the practice guideline for traditional Chinese medicine preventive treatment on insomnia disorder
Source: Front Psychiatry. 2025 Apr 16;16:1475904. doi: 10.3389/fpsyt.2025.1475904 (PMC12041864; doi:10.3389/fpsyt.2025.1475904)
Supplement: Supplementary file 6 [file DataSheet6.pdf]

Supplementary material 6. English Search strategies

#1 "Sleep Initiation and Maintenance Disorders"[MeSH Terms] OR "Disorders of Initiating and Maintaining Sleep"[Title/Abstract]OR "DIMS (Disorders of Initiating and Maintaining Sleep)"[Title/Abstract] OR "Early Awakening"[Title/Abstract]OR "Awakening, Early"[Title/Abstract]OR "Nonorganic Insomnia"[Title/Abstract]OR "Insomnia, Nonorganic"[Title/Abstract]OR "Primary Insomnia"[Title/Abstract]OR "Insomnia, Primary"[Title/Abstract]OR"Transient Insomnia"[Title/Abstract]

#2 "Insomnia, Transient"[Title/Abstract]OR "Rebound Insomnia"[Title/Abstract]OR "Insomnia, Rebound"[Title/Abstract]OR "Secondary Insomnia"[Title/Abstract]OR "Insomnia, Secondary"[Title/Abstract]OR "Sleep Initiation Dysfunction"[Title/Abstract]OR "Dysfunction, Sleep Initiation"[Title/Abstract]OR "Dysfunctions, Sleep Initiation"[Title/Abstract]OR "Sleep Initiation Dysfunctions"[Title/Abstract]

#3 "Sleeplessness"[Title/Abstract]OR "Insomnia Disorder"[Title/Abstract]OR "Insomnia Disorders"[Title/Abstract]OR "Insomnia"[Title/Abstract]OR "Insomnias"[Title/Abstract]OR "Chronic Insomnia"[Title/Abstract]OR "Insomnia, Chronic"[Title/Abstract]OR "Psychophysiological Insomnia"[Title/Abstract]OR "Insomnia, Psychophysiological"[Title/Abstract]

#4 "Sleep Wake Disorders"[Title/Abstract]OR "Dyssomnias"[Title/Abstract]OR "Sleep Disorders, Intrinsic"[Title/Abstract]OR "Insomnia, Fatal Familial"[Title/Abstract]

#5 "Co-morbid Insomnia and Obstructive Sleep Apnea"[Title/Abstract] OR "Obstructive Sleep Apnea"[Title/Abstract] OR "Syndrome, Sleep Apnea, Obstructive"[Title/Abstract] OR "Sleep Apnea Hypopnea Syndrome"[Title/Abstract] OR "Restless Legs Syndrome"[Title/Abstract] OR "Willis Ekbom Syndrome"[Title/Abstract] OR "Willis Ekbom Disease"[Title/Abstract] OR "Periodic Limb Movement Disorder"[Title/Abstract] OR "Nocturnal Myoclonus Syndrome"[Title/Abstract] OR "Sleep-Related Periodic Leg Movements, Excessive"[Title/Abstract] OR "Sleep Myoclonus Syndrome"[Title/Abstract]OR "Narcolepsy"[Title/Abstract] OR "Gelineau Syndrome"[Title/Abstract] OR "Paroxysmal Sleep"[Title/Abstract] OR "Sleep Paralysis"[Title/Abstract] OR "Chronobiology Disorders"[Title/Abstract] OR "Circadian Rhythm Disorder"[Title/Abstract]

#6 #1 OR #2 OR #3 OR #4 OR #5

#7 "Acupuncture"[MeSH Terms] OR " Acupuncture Therapy"[MeSH Terms] OR "Acupuncture Treatment"[Title/Abstract] OR "Electroacupuncture"[Title/Abstract]OR "Umbilical acupuncture"[Title/Abstract] OR "Ear Acupuncture"[Title/Abstract] OR "Auricular Acupuncture"[Title/Abstract] OR "scalp acupuncture"[Title/Abstract] OR "abdominal acupuncture"[Title/Abstract] OR "Eye Acupuncture"[Title/Abstract] OR "Buccal acupuncture"[Title/Abstract] OR "arm acupuncture"[Title/Abstract] OR "wrist-ankle acupuncture"[Title/Abstract] OR "body acupuncture"[Title/Abstract]

#8 "Blade acupuncture"[Title/Abstract] OR "filiform needle"[Title/Abstract]OR"thumbtack needle"[Title/Abstract] OR " fire needle"[Title/Abstract] OR "bee acupuncture "[Title/Abstract] OR " plum blossom needle"[Title/Abstract] OR " silver needle"[Title/Abstract]

#9 #7 OR #8

#10 #6 AND #9
